# Frontoparietal structural properties mediate adult life span differences in executive function

**DOI:** 10.1038/s41598-020-66083-w

**Published:** 2020-06-03

**Authors:** Zai-Fu Yao, Meng-Heng Yang, Kai Hwang, Shulan Hsieh

**Affiliations:** 1 Brain and Cognition, Department of Psychology, University of Amsterdam, Amsterdam, The Netherlands; 2 Department of Psychology, National Cheng Kung University, Tainan, Taiwan; 3 Department of Psychological and Brain Sciences, University of Iowa, Iowa, USA; 4 Iowa Neuroscience Institute, University of Iowa, Iowa, USA; 5 Institue of Allied Health Sciences, National Cheng Kung University, Tainan, Taiwan; 6 Department and Institute of Public Health, National Cheng Kung University, Tainan, Taiwan

**Keywords:** Cognitive ageing, Cognitive neuroscience, Human behaviour

## Abstract

Executive function (EF) refers to a set of cognitive functions that support goal-directed behaviors. Recent findings have suggested that the frontoparietal network (FPN) subserves neural processes that are related to EF. However, the FPN structural and functional network properties that mediate age-related differences in EF components remain unclear. To this end, we used three experimental tasks to test the component processes of EF based on Miyake and Friedman’s model: one common EF component process (incorporating inhibition, shifting, and updating) and two specific EF component processes (shifting and updating). We recruited 126 healthy participants (65 females; 20 to 78 years old) who underwent both structural and functional MRI scanning. We tested a mediation path model of three structural and functional properties of the FPN (i.e., gray matter volume, white matter fractional anisotropy, and intra/internetwork functional connectivity) as mediators of age-related differences in the three EF components. The results indicated that age-related common EF component differences are mediated by regional gray matter volume changes in both hemispheres of the frontal lobe, which suggests that structural changes in the frontal lobe may have an indirect influence on age-related general elements of EF. These findings suggest that the FPN mediates age-related differences in specific components of EF.

## Introduction

Aging is associated with the deterioration of executive function (EF)^1,2^. EF (also known as cognitive control) describes processes needed for goal-directed behaviors and can be partitioned into several component functions^3,4^. An influential framework, the Unity and Diversity model introduced by Miyake *et al*. (2000)^4^, identified three dissociable components of EF, including updating-specific EF (temporarily keeping information accessible for processing), shifting-specific EF (switching between different tasks), and inhibition-specific EF (withholding intended responses) components. These three EF components are moderately correlated (i.e., unity), suggesting that a common process supports all EFs but are also divisible (i.e., diversity) and can be partitioned into subcomponents, which indicates that each specific component EF engages a distinct neurocognitive process^3,4^. Further analyses by Miyake’s group revealed that variances in inhibition task performance can be explained mostly by a “common” EF factor that also explains a large proportion of performance variances in other EF tasks^3,5,6^. This suggests that there is likely no specific EF component for inhibition.

In regard to aging, studies have found that behavioral performances on tasks that recruit these component EFs decline with age. For instance, aging has been associated with decreased working memory capacity^7,8^, prolonged task-switching cost^9,10^, and decreased motor inhibition^11,12^. However, there are also individual differences in age-related declines in EF. Some older adults show mild cognitive decline, while others display more significant cognitive impairment than others of the same age^13^. These individual differences suggest that other factors mediate the relationship between aging and EF and that these mediating factors may predict individual differences. One candidate mediating factor is brain structural properties. Elderly adults show increased variability in cortical thickness^14–16^, and cognitive decline emerges after the manifestation of a substantial pathology of the white matter (WM)^17,18^. Another candidate factor is the functional network organization. The ability of one brain region to transmit information to another distant brain region can be estimated by the integrity of structural connectivity^19^, which itself can be estimated by fractional anisotropy (FA). The strength of functional connectivity can be estimated by the correlation between signals recorded from different brain regions. Evidence has suggested that both structural connectivity and functional connectivity mediate age-related differences in EF. For example, functional connectivity between the putamen and occipitoparietal regions mediates age-related declines in Stroop task performance^20^, and the thickness of cortical regions and the averaged FA values of white matter regions mediate the relationship between age and EF^21^. A recent study also found that functional connectivity between sensorimotor regions mediates age-related differences in composite measures across digit-symbol coding, Stroop, and verbal fluency tasks^22^.

EF tasks are known to modulate frontal and parietal activities^23–25^. Specifically, studies that putatively test specific EFs, such as updating-specific EF^26,27^, shifting-specific EF^28–32^, and inhibitory-specific EF^27,33,34^, have consistently reported increased frontal and/or parietal activities. This reliable relationship between EF and frontoparietal activities strongly suggests that frontoparietal properties may mediate age-related declines in EF. The findings from one of our earlier studies support this notion^35^. In this earlier study^35^, we examined the relationships among multimodal neuroimaging measures, age, and component EFs based on Miyake’s Unity and Diversity model. Specifically, we used a joint independent component analysis (jICA) to derive, across the whole brain, joined multimodal components that integrated information from gray matter (GM) volume, FA (a measure of structural connectivity), and amplitude of low-frequency fluctuation (a measure of the spontaneous neural activity in specific regions) in a given region. We then examined the relationships of these measures between age and EFs. The results showed significant age-related differences in all EF estimates (common EF, updating-specific EF, and shifting-specific EF). These age-related differences were associated with joined multimodal components that encompassed the frontal and parietal regions.

While our earlier study^35^ found significant correlations between frontoparietal properties and EF and between EF and age, we did not explicitly test for whether frontoparietal properties mediated age-related declines in EF. Furthermore, jICA cannot separate the specific contributions from GM volume, structural connectivity, and functional connectivity. We do not know if all multimodal imaging measures share the same mediation effect on age-related changes in EF or if each individual imaging property has differential mediating effects. As such, the current study aimed to address these unanswered questions from results reported in Yang and colleagues (2019)^35^, using mediation models to test if and which structural and network properties of the frontoparietal cortices mediate age-related changes in EF. In addition, although there are a few multimodal mediation studies examining the effects of age on tasks measuring EF^36–38^, to the best of our knowledge, none of these prior studies were based on Miyake’s EF model. For example, Hedden *et al*.^21^ incorporated multimodal imaging measures in a mediation model, but they probed only one aspect of EF, such as memory. Therefore, we sought to examine whether the aforementioned age-related declines in structural and functional brain changes are similarly dissociable depending upon the EF component process being tested.

In this study, we used the Unity and Diversity model to measure behavioral performances in three EF tasks (i.e., the task-switching paradigm, the 2-back task, and the stop-signal task) and derived common EF (general EF component incorporating inhibition, shifting, and updating), the shifting-specific EF (as measured by performance in the task-switching paradigm), and the updating-specific EF (as measured by performance in the 2-back task) components^3,4,6,39^. To validate the common EF component, the inhibition-specific EF component was also calculated to examine the association among EF components. We tested a mediation path model^21,40,41^ that included age as the independent variable, EF measures as the dependent variables, and the following multimodal neuroimaging measurements as mediators: structural property (GM volume), structural connectivity (WM integrity, as measured by FA), and functional network properties (as measured by graph-theoretic inter- and intra-network functional connectivity metrics; details are described in the Methods section). The mediation model enabled us to test whether the combined indirect effects of the total potential mediators controlled both collinearities among variables and mediation effects, meaning that any significant indirect effects are independent^42^. Furthermore, we were particularly interested in the specific indirect effects that the brain imaging measures had on EF components. Our goals were to determine which structural and network properties of the frontoparietal cortices mediate age-related differences in EFs. Specifically, we investigated whether age-related changes in structural and functional brain network properties in the frontoparietal cortices are similar across EF component processes or whether changes with aging are specific to the EF component process being tested.

## Results

### The relationship between age and behavioral results

#### Task switching paradigm: switch cost

The average switch cost was 78.69 ± 112.83 ms. We observed a significant correlation between switch cost and age (r = −0.20, p = 0.025). The mean accuracy rate for all repeat trials in the single blocks was 98.01 ± 1.52%; for repeat trials in the mixed blocks, it was 95.82 ± 2.32%, while for switch trials in the mixed blocks, it was 92.75 ± 3.21%.

#### 2-back task: d’

The mean accuracy in the 2-back task was 80.71 ± 12.73%. The results of the n-back tasks are reported based on the signal detection theory. A significant correlation was observed between age and the 2-back d’ (r = 0.41; p = 3.00E-06), suggesting poorer 2-back task performance with increasing age.

#### Stop-signal task: Stop-signal reaction time (SSRT)

The mean stop inhibition rate (stop success rate) was 53.38 ± 0.15%, and the mean SSRT was 241.28 ± 113.29 ms. SSRTs were significantly correlated with age (r = 0.37; p = 2.496E-05), suggesting a poorer stopping performance with increasing age. SSRTs were not correlated with the mean RT in the correct go trials (r = −0.09; go-trial RT: 658.61 ± 12.13 ms; mean accuracy rate: 90.39 ± 3.3%), which is consistent with the “horse-race” model^43^ that assumes process independence between go trials and stop trials.

#### Executive function components based on Miyake *et al*. ’s (2000) model: Age association with common EF and specific (shifting and updating-specific) EF

**Figure 1:**
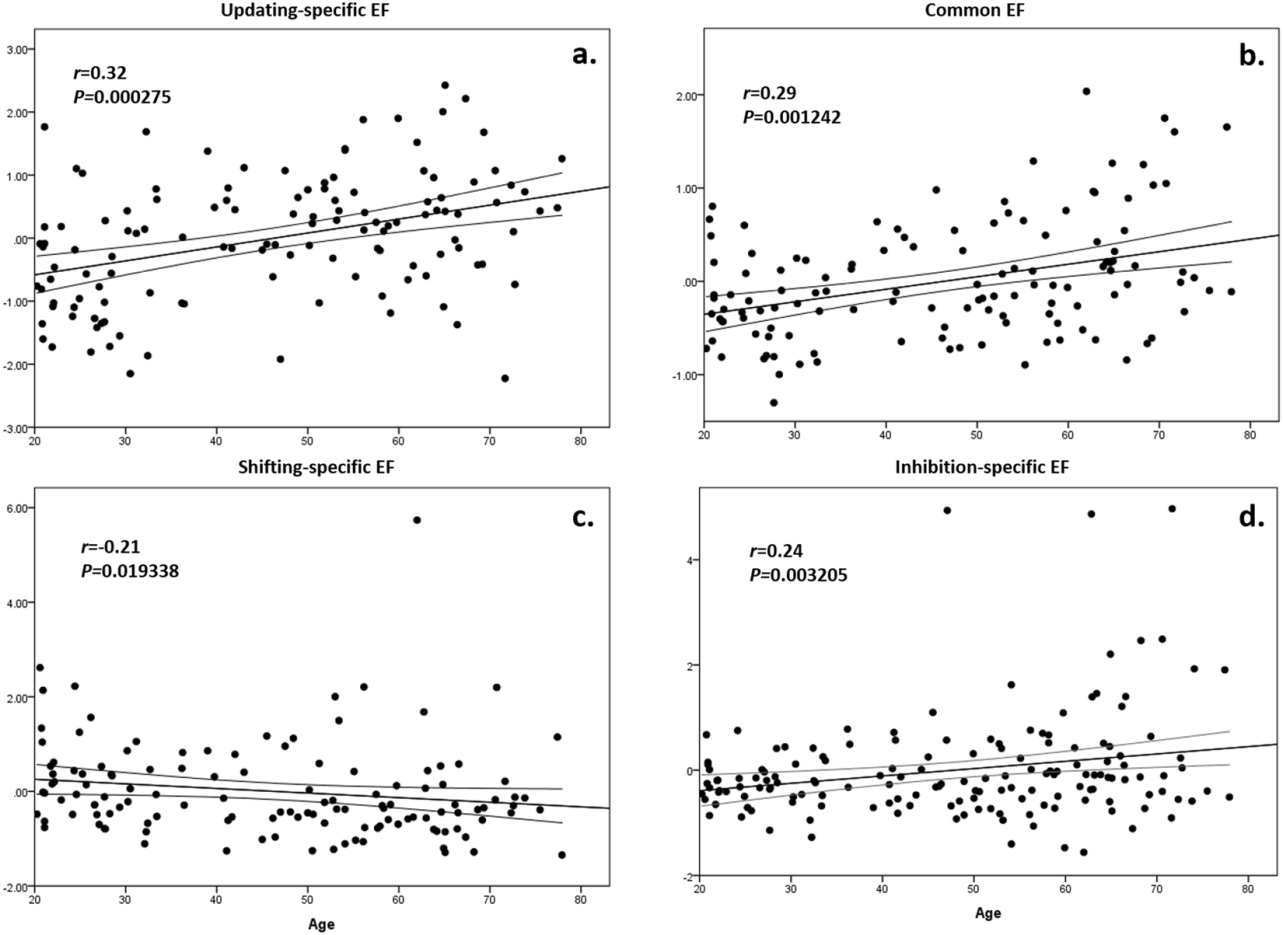
Scatterplots with lines of best fit (±CI) showing age (x-axis) against each of the common and specific components of executive function (y-axis): (**a**) updating-specific EF; (**b**) common EF; (**c**) shifting-specific EF; (**d**) inhibition-specific EF; r denotes pearson’s r value (with p value); CI = confidence interval; EF = executive function.

Based on Miyake *et al*. ’s (2000) model, we calculated one common and two specific EF components, that is, shifting and updating. The results indicated that these EF components are significantly correlated with age (common EF: r = 0.29, p = 1.242E-03; updating-specific EF: r = 0.32, p = 2.750E-04; shifting-specific EF: r = −0.21, p = 1.934E-02) (see Fig. 1a-c). To validate the common EF component, the inhibition-specific EF component was also calculated and reported (see Fig. 1d). Correlation analysis among EF components found that the common EF component was significantly correlated with shifting-specific EF (r = 0.555, p < 0.0001), updating-specific EF (r = 0.46, p < 0.0001), and inhibition-specific EF (r = 0.559, p < 0.0001) components. There was neither a significant correlation between the updating-specific EF and the shifting-specific EF components (r = −0.28, p = 0.757) nor a correlation between the inhibition-specific EF and shifting-specific EF components (r = 0.006, p = 0.95). However, a significant correlation was found between the inhibition-specific EF and updating-specific EF components (r = −0.228, p = 0.011).

### Age differences in brain measures across different modalities

**Figure 2:**
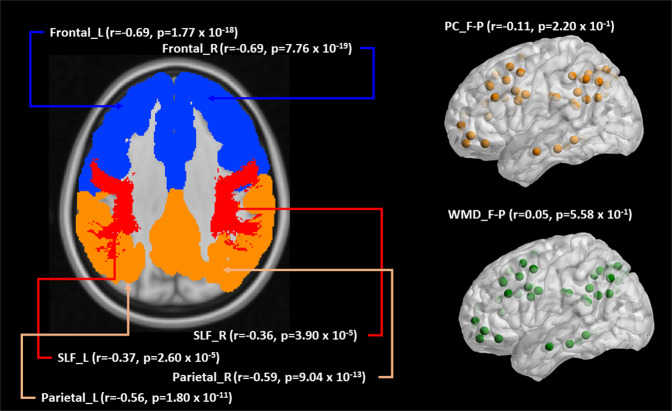
Correlation between age and all brain measures. SLF = superior longitudinal fasciculus; F-P = fronto-parietal lobules; L = left; R = right; PC = participation coefficient; WMD = within-module degree. Bonferroni correction sets the significance level of 0.00625 to correct for the false- discovery bias inherent in multiple-comparison testing.

Age was significantly correlated with four brain measures in two modalities (GM volume, WM FA), as displayed in Fig. 2. There was no significant association between age and either PC or WMD scores within frontal and parietal lobules. All other correlations between age and brain measures were significant negative correlations (r range: −0.36 ~ −0.69), indicating that increased age was associated with lower values of WM FA and GM volumes.

### Brain measures as mediators of age-cognition relationships

**Figure 3:**
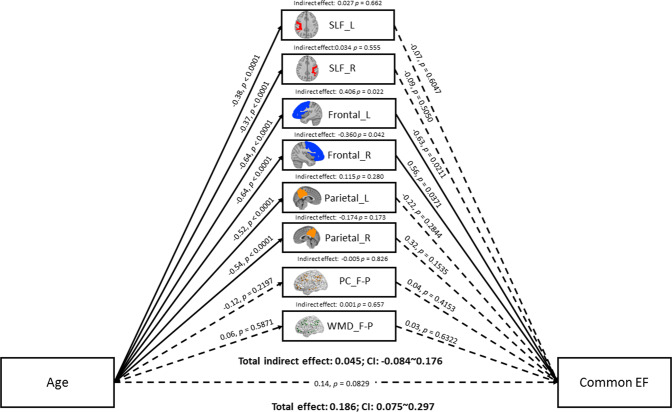
Mediation model of age, brain measures, and common EF. Solid lines indicate significant paths (p < 0.05), and the dashed line indicates non-significant paths. Path values indicate standardized beta weights and p values. CI indicates the 5000 samples bootstrapped with 95% confidence intervals for the indirect and total effects. SLF = superior longitudinal fasciculus; L = left; R = right; F-P = fronto-parietal lobules; PC = participation coefficient; WMD = within-module degree.

**Figure 4:**
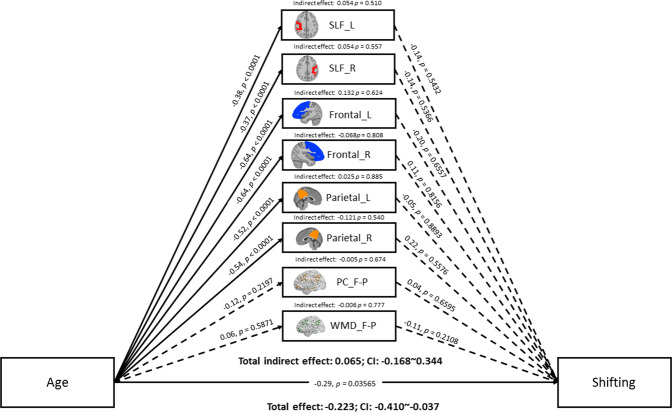
Mediation model of age, brain measures, and shifting-specific EF. Solid lines indicate significant paths (p < 0.05), and the dashed line indicates non-significant paths. Path values indicate standardized beta weights and p values. CI indicates the 5000 samples bootstrapped with 95% confidence intervals for the indirect and total effects. SLF = superior longitudinal fasciculus; L = left; R = right; F-P = fronto-parietal lobules; PC = participation coefficient; WMD = within-module degree.

**Figure 5:**
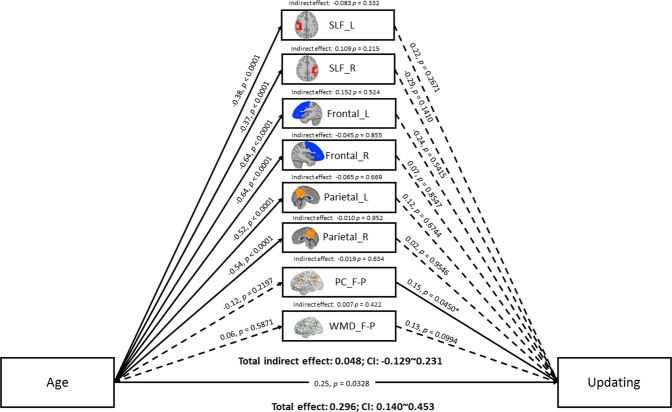
Mediation model of age, brain measures, and updating-specific EF. Solid lines indicate significant paths (p < 0.05), and the dashed line indicates non-significant paths. Path values indicate standardized beta weights and p values. CI indicates the 5000 samples bootstrapped with 95% confidence intervals for the indirect and total effects. SLF = superior longitudinal fasciculus; L = left; R = right; F-P = fronto-parietal lobules; PC = participation coefficient; WMD = within-module degree.

We tested a parallel mediation model of structural and functional brain measures within the frontal and parietal regions mediating the relationship between age and the unity and diversity EF components of Miyake and Friedman. The results of the mediation analyses in terms of each EF component are summarized in Figs. 3–5. The results of the mediation analyses showed no significant total indirect effect of the brain-mediation model on all age-related EF components. Specifically, the 95% bias-corrected confidence interval based on 5000 bootstrap samples showed that the total indirect effect did not reach significance with the common EF (r = 0.05, p = 0.50 [95% CI = −0.0889~0.1810]), shifting-specific EF (r = 0.07, p = 0.61 [95% CI = −0.1689~0.3354]), and updating-specific EF (r = 0.05, p = 0.60 [95% CI = −0.1255~0.2361) components. Nonetheless, a significant specific indirect effect on the common EF component was found in GM volumes of the left (r = 0.41, p = 0.02 [95% CI = 0.1060~0.7988]) and right (r = −0.36, p = 0.04 [95% CI = −0.7787~ −0.0676]) frontal lobules.

## Discussion

In this study, we aimed to determine which functional and structural brain properties within the frontal and parietal regions mediate age-related differences in common and specific components of EF. Given the substantial evidence that has implicated the frontoparietal cortices in EF^23–25,35^, we focused specifically on the properties of the frontoparietal cortices. We selected our EF tasks based on Miyake’s model, which distinguishes three EF components^3,4,6,44,45^: common EF, updating-specific EF, and shifting-specific EF. This study attempted to extend our recent study^35^ showing significant correlations among EF, the frontoparietal cortices, and age. Specifically, we sought to determine whether the structural and network properties of the frontoparietal cortices mediate age-related changes in EF and, further, to determine whether the mediation effect was selective for a specific EF component or whether it was a generalized pattern for all EF components.

Previous studies have reported that EF deteriorates with age^46–48^. Consistent with the multifaceted nature of EF, we found that all components of Miyake’s EF model exhibited age-related declines across the adult life span. Of all EF components, we found that only the common EF component was significantly mediated by imaging measures of GM volume in the frontal lobe of both hemispheres. Specifically, the common EF component was not only related to response inhibition, based on Miyake’s EF model^3,6^ but also represented a generalized mechanism that contributes to inhibition, updating and shifting EF. Common EF is interpreted as the ability to maintain task goals and use goal-related information to bias lower-level processing^3,49^ and is likely an underlying common and required process that supports general elements of EF components. Based on Miyake’s model, it is probable that the common EF components are intercorrelated among each other. Correlation results from our study also showed that the common EF component significantly correlated with other EF components (i.e., inhibition-specific, updating-specific, and shifting-specific), indicating that the common EF component supports shared abilities among EF processes. The correlation among EF components means that the components possibly share similar relationships that change at the same time but do not necessarily interact with the same sequential relationships^50^. Therefore, both variables interact with the same variable, the identify of which remains unknown based on correlation analyses. The inclusion of a mediator necessarily increased the minimum distance between both variables, enabling the mediated effects to be investigated as a directionality relationship between variables^51^. The findings from our study, with the application of mediation, suggested that an age-related difference in the general elements of EF (i.e., a common EF component) may be influenced by GM atrophy, particularly the density of cell bodies in both hemispheres of the frontal region.

The associations between GM imaging measures and age-related cognitive performance changes have been evident in previous studies with large sample sizes^52–55^, possibly indicating the early sign of age-related decline. For example, a recent study^55^ found that an age-related difference in EF was associated with GM volume but not thickness. These findings found GM volume loss in widespread brain regions was associated with diverse tasks that measure EF performance^52,54,55^. These observations, coupled with our findings, indicate that associations among EF components and the frontoparietal cortices may exist. Specifically, frontal GM volume reductions play a role in mediating changes in age-related general elements of EF. These putative mechanisms also clarify our earlier work^35^, which found that joint components in the frontal regions contributed to the age-related common cognitive control construct.

Unlike our earlier work^35^ using a whole-brain data-driven approach that fused multimodal brain imaging measures to search for potential multimodal markers of age-associated EF component decline, this study examined the role of structural and network metrics in frontoparietal cortices that mediate age-associated changes in EF components. As previous technical guidelines have suggested^56–59^, mediation is better used to test whether a hypothesis explains the indirect effect of an independent variable on a dependent variable. Moreover, the investigation of multimodal brain networks in the frontoparietal cortices derived from brain imaging data allows us to investigate the topological organization of the human brain^60^. Nonetheless, the results revealed no specific indirect effect of WM integrity and the functional network properties (i.e., internetwork and intranetwork connection strength) of the frontoparietal cortices, suggesting that differences in age-associated EF components may not be influenced by age through WM integrity and the functional network properties of the frontoparietal cortices. Furthermore, there were no significant mediation effects of multimodal imaging measures in the frontoparietal cortices in terms of the shifting and updating-specific EF components, reflecting the diversity in EF component development in the adulthood lifespan. Based on the Unity and Diversity model introduced by Miyake *et al*. (2000), these EF components (i.e., shifting and updating-specific EF) were distinct from the common EF component. Please note, although we did not observe that WM integrity and functional network properties mediated age-related decline in common EF, this does not suggest that they did not play any role. Here, we can only infer that GM played a more critical role than the other two imaging properties based on the results of mediation models, and this finding also adds additional information to our earlier findings.

In summary, our study indicates that GM imaging measures of frontal lobules mediate age-related differences in common EF but not in shifting-specific and updating-specific EF. Furthermore, the left frontal GM volume explains an age-related difference in the common EF component. Our study successfully detected the relationships among three different imaging metrics and the multifaceted components of EF. Our approach can be used to explore other multivariate relationships between brain imaging measures and cognition. The findings from this study suggest that changes in GM volume may be attributed to age-related EF decline, as changes in GM volume reflect the loss of cell density. EF comprises diverse cognitive skills that are crucial for daily functioning. Our findings go beyond those of previous investigations of age-related cognitive decline. We demonstrate that the inclusion of different brain imaging measures may help to reveal—years before the symptoms appear—markers for the early detection and identification of those who are at risk of age-related cognitive decline. Moreover, future studies can utilize multimodal brain imaging measures that may offer more accuracy in terms of predicting an individual’s cognitive performance.

## Methods

### Participants

**Figure 6:**
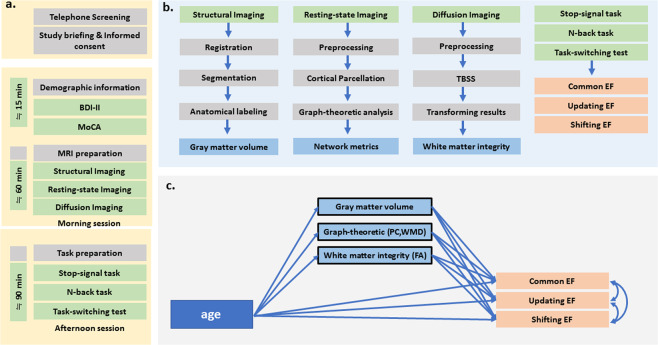
Schematic of experimental procedure and pipeline analysis. (**a**) Study design for performing experiments. (**b**) Pipeline analysis for brain imaging measures. (**c**) Pipeline analysis for mediation model.

We recruited 183 participants from Tainan city, Taiwan, by using online advertisements and bulletin boards. Participants were the same as those reported in our earlier work^35^. Before formal testing, all participants completed cognitive assessments, including the Montreal Cognitive Assessment (MoCA) to screen for cognitive impairment^61^ and the Beck Depression Inventory-II (BDI-II^62^) to screen for depression. Participants were excluded from subsequent analysis if their MoCA scores were ≤25 (n = 30) or if their BDI-II scores were ≥14 (n = 7). To mitigate potential confounds, we added gender, education, and BDI-II scores as covariate variables for the following analysis. In addition, sixteen participants were excluded due to MRI technical problems (e.g., head-motion-induced artifacts) or failure to complete the entire experiment (e.g., feeling uncomfortable during the long period of scanning and asking to leave the scanning room). After the exclusion of those who failed to meet the criteria, the remaining 126 right-handed participants reported no prior history of mental disorders or neurological disease. The mean age (± standard deviation, SD) of the 126 participants (74 females) was 46.32 ± 17.40 years (range 20–78). The mean BDI-II score was 5.14 ± 0.32. See Table 1 for participants’ age range distribution and demographic information. The experimental design procedure is shown in Fig. 6.

**Table 1:** Demographic information and behavioral assessment scores of 126 participants.

age group (year)	n	Female (% of total sample)	MoCA Mean(SD)	Education Mean(SD)	BDI_II Mean(SD)	age Mean(SD)
20–30	34	41.18%	28.59(±0.96)	16.26(±1.29)	4.97(±3.66)	24.27(±2.93)
30.01–40	15	40.00%	28.07(±1.33)	15.93(±1.79)	6.00(±4.57)	33.72(±3.09)
40.01–50	15	46.67%	27.93(±1.10)	13.89(±2.56)	5.73(±3.51)	44.85(±2.95)
50.01–60	28	64.29%	27.57(±1.23)	14.61(±2.57)	4.82(±4.11)	54.84(±3.15)
60.01–70	24	58.33%	27.79(±1.35)	14.00(±2.47)	4.29(±4.31)	65.08(±2.37)
70.01–80	10	20.00%	27.10(±0.99)	14.20(±2.53)	3.10(±3.11)	73.53(±2.61)

Note: SD = standard deviation; MoCA = Montreal Cognitive Assessment; BDI-II = Beck Depression Inventory II.

The research protocol followed the Research Ethics rules of research of National Cheng Kung University under the governance framework of the Human Research Ethics Committee and with respect to the Declaration of Helsinki to protect the participants’ right to codetermination. Participants provided their informed consent before joining the study and were awarded 1,500 NTD after completing the study.

### Cognitive tasks

#### Task-switching paradigm

Task-switching abilities were measured by a paradigm modified from Karayanidis *et al*. ’s study^63^. The procedure was identical to that of our earlier work (for details, refer to Yang *et al*.^35^). The visual stimuli were displayed using Presentation software on a 17-inch monitor with a 1024 × 768 resolution. There were two cuing conditions, namely, informative and noninformative. The informative cuing condition (i.e., informed task conditions contained two color cues that informed of the forthcoming task; hot (e.g., orange or red) and cold (e.g., green or blue) were associated with a letter or number classification task, respectively). To reduce the effects of repeatedly presenting physically identical cues, these color cues were not repeated in successive trials. The target stimuli were presented in white, whereas the background was presented in black. For the noninformative cuing conditions (i.e., noninformed task conditions), the cues were presented in gray (the background was in black), which provided no inforrmation regarding the forthcoming task type. The target stimuli were presented in either hot (red or orange) or cold (blue or green) colors (similar to the informative cue colors). In this study, we restricted the analyses to informative cue conditions so that we could focus on pure switch cost effects.

Specifically, the stimuli were composed of an incongruently mapped bivalent Chinese letter–Arabic number pair or a neutral pair. Chinese letters were composed of eight Chinese letters from the Ten Celestial Stem system (i.e., Tiangan). Tiangan is a Chinese system of ordinals, which is the literal meaning of the Chinese counting system that is similar to the English alphabet. The participants were instructed to respond using either the left or right index finger mapped to first-half/second-half or odd/even for Chinese-letter and Arabic-number tasks, respectively. Both cue-task mapping and hand-task mapping conditions were counterbalanced. The probability of a switch between conditions was set at 50%, with no more than four mixed-repeat or switch trials in succession during mixed-task blocks.

Each participant sat in a chair and stared at the center of a computer screen that had been placed at a distance of 100 cm. A cue and a target were presented in each trial. The cue stimuli were presented for 600 ms, followed by a cue-target interval of 1000 ms. The target was presented either for 5000 ms or until a response was given. The interval between a given response to the next target was 1600 ms. The participants were told to respond by pressing the button as quickly and as accurately as possible. Each error was responded to with immediate auditory feedback, and the next trial was postponed by 1000 ms. Performance measures, including mean reaction time (RT) and the error rate, were reported after each block of trials was completed. The participants first practiced six types of blocks: (1) one single-task block of 16 trials with number stimuli; (2) one single-task block consisting of 16 trials with Chinese letters; (3) two blocks of 32 trials with a mixed informed task per block; and (4) two mixed blocks of 32 trials with noninformed task per block. The experiment consisted of 12 blocks: (1) two single-task (Arabic number’s odd/even task) blocks; (2) two single-task (Chinese-letter’s first-half/second-half task) blocks; (3) four mixed informed task blocks; and (4) four mixed noninformed task blocks. Each block consisted of 70 trials. The entire experiment lasted for 30 to 40 minutes depending on individual response differences. We calculated the switch cost by subtracting the average RT of the repeat trials in the mixed-task blocks from the average RT of the switch trials in the mixed-task blocks^64^.

#### 2-back task

The participants were asked to complete a 2-back working memory task from Jaeggi *et al*.’s study^65^. The procedure was identical to that from our earlier work (for details, refer to Yang *et al*.^35^). In this task, the stimuli were presented within a 3-by-3 grid in each trial. One of the grid squares was randomly assigned to be filled with blue. The grid square with blue appeared in a random position within the 3-by-3 grid. For the 2-back test, participants were instructed to memorize the position of the blue grid square shown in the previous two trials and to compare it to the position of the blue grid square in the current trial. If the blue grid square has appeared in the same location, participants pressed the “F” button using their left index finger. If the blue grid square has appeared in a different location, participants pressed the “J” button using their right index finger. The grid stimulus was presented for 500 ms and was followed by an interstimulus interval (ISI) for 2000 ms. The participants were asked to respond before the next trial. The participants completed one practice block with feedback and then completed three formal blocks (21 trials per block). The entire experiment lasted for 30 to 40 minutes.

We calculated performance sensitivity (*d’*) as an index of 2-back task performance^66^, which was based on the hit rate (H) and false-alarm (F) rate, using the formula *d’* = *Z*(H) − *Z*(F) (Z denotes the z score of the normal distribution.). This sensitivity index, d’, distinguished better performance from poorer performance by discriminating targets from nontargets during the performance of the task. To better delineate the relationship between age and brain structure/function, we transformed d’ into negative values to suggest that a higher d’ value (i.e., less negative) would indicate worse performance. Thus, behavioral performance showed similar trends to ease interpretation.

#### Stop-signal task

The stop-signal task was a modified version of Logan and Cowan’s paradigm^67^. The procedure was identical to that from our earlier work (for details, refer to Yang *et al*.^35^). The participants were asked to stare at the visual stimulus presented on the display screen and respond to the presented target “O” or “X” by using their left or right index fingers to press either the “z” or “/” button, respectively. The background of the screen was white, while the target stimulus was presented in black. The target stimulus “O” or “X” (2 cm in size and at a visual angle of 0.64°) was displayed in the middle of the screen for 100 ms. The participants were told to react to the stimulus as quickly and as accurately as possible. An auditory stimulus (i.e., a “beep” sound of 968 Hz) lasting for 100 ms could be delivered during the task and served as a “stop” signal. All participants were instructed to ignore this sound in the first practice session so that they could familiarize themselves with the process of responding to the stimuli as quickly and accurately as possible. During the second practice session, the participants were told to immediately cease their intended action when they heard the “beep” sound (auditory stop signal). During the practice session, the auditory stop signal was delivered at a frequency of 500 Hz for 300 ms after the onset of the presented stimulus. Importantly, the participants were informed to not hold their responses while they waited for the auditory stop signal. The formal session, which started after the practice session, comprised five blocks of 140 trials consisting of 40 stop trials and 100 go trials. We varied the stop-signal delay (SSD) according to participant responses during the stop trials. In each stop trial, the SSD was randomly chosen from one of two interleaved staircases, each starting with a value of 150 or 350 ms. If participants successfully withheld their motor response, then the SSD of the next stop trial would increase by 50 ms. Conversely, if participants did not withheld their response, the SSD would decrease by 50 ms in the next stop trial. The SSD ranged from 0 to 800 ms. The purpose of the staircase procedure was to ensure that the successful stopping rate was maintained at at least 50% of all stop trials. The interstimulus interval (ISI) ranged from 1300 to 4800 ms. The total duration for completing this task was 30 minutes. The stop-signal reaction time (SSRT) was calculated by subtracting the median SSD from the median RT of the go trials^43^. The argument could also be made to employ other commonly used methods such as the integration method. However, the integration method assumes that SSRT is a constant, which may be more susceptible to violations of the assumptions of the independent horse-race model than other estimation methods^68,69^.

### Computing common and specific executive function components

Based on Miyake and Friedman’s (2012) procedures, we computed the Z value for each task performance measure (i.e., updating [monitoring and changing working memory content], shifting [flexible changes between task sets or goals], and inhibition [overriding withholding habitual or prepotent responses]) of each participant. To validate the common EF component, the inhibition-specific EF component was also calculated in a manner similar to other specific EF components (i.e., updating- and shifting-specific components) by regressing out the SSRT against switching cost and 2-back d’. Correlation analyses among the EF-specific components was also conducted. These three z scores were averaged for each participant’s task performances (i.e., stop-signal task, task-switching paradigm, and 2-back task) to create a composite score reflecting a common (i.e., unity) executive function component (common EF component) and two specific executive function components (i.e., updating and shifting-specific EF components). Accordingly, the function of inhibition was then seen as “common” EF and has been shown to influence performance on all three EF tasks^3,6^. For each specific (i.e., diversity) EF component, we regressed out the performance of the targeted task against the other two tasks’ performance, thereby yielding a specific residual. Specifically, for a shifting-specific EF, we regressed out the switching cost against SSRT and 2-back d’, while for the updating-specific EF, we regressed out the 2-back d’ against the switch cost and SSRT.

### Image acquisition and processing

The image acquisition parameters were identical to those in our earlier work (for details, refer to Yang *et al*.^35^). A GE MR750 3 T scanner (GE Healthcare, Waukesha, WI, USA) installed in the Mind Research and Imaging Center at National Cheng Kung University (NCKU) was used to acquire all brain imaging data. High-spatial-resolution T1-weighted images were scanned with a fast spoiled gradient echo (fast SPGR) sequence (166 axial slices; repetition time (TR): 7.6 ms; echo time (TE): 3.3 ms; flip angle: 12°; field of view (FOV): 22.4 × 22.4 cm^2^; matrices: 224 × 224; slice thickness: 1 mm). The entire scanning time (to completion) lasted 218 seconds.

We scanned the resting-state functional images using a gradient-echo planar imaging (EPI) pulse sequence (TR/TE/flip angle, 2000 ms/30 ms/77°; matrices, 64 × 64; FOV, 22 × 22 cm^2^; slice thickness, 4 mm; voxel size, 3.4375 × 3.4375 × 4 mm). The total scan time was 490 seconds. We discarded the first five dummy scans to mitigate T1 equilibrium effects. During the session involving resting-state image scanning, we asked the participants to stay awake with their eyes open and to fix their gaze on the white cross sign on the screen. The entire scanning session lasted for 8 minutes. This procedure reflects recent evidence^70^ showing that eyes open and fixated produce more reliable results for controlling experimental variability across participants.

Diffusion tensor imaging (DTI) was carried out using a spin-echo echo planar sequence (TR/TE = 5500 ms/62~64 ms, 50 directions with b = 1000 s/mm^2^, 100 × 100 matrices, slice thickness = 2.5 mm, voxel size = 2.5 × 2.5 × 2.5 mm, number of slices = 50, FOV = 25 cm, NEX = 3). The total scan time was 924 seconds. A reversed-phaseencoding DTI (TR/TE = 5500 ms/62~64 ms, 6 directions with b = 1000 s/mm^2^, 100 × 100 matrices, slice thickness = 2.5 mm, voxel size = 2.5 × 2.5 × 2.5 mm, number of slices = 50, FOV = 25 cm, NEX = 3) was also acquired for top-up correction in the DTI preprocessing. The total scan time was 198 seconds. The acquisition parameters for the reversed-phase-encoding DTI were identical to the DTI, with the only difference being the number of directions as six. The reason for choosing fewer numbers of reversed-phase-encoded directions was to avoid motion artifacts induced by participant discomfort resulting from long imaging times in the elderly participants.

### DTI processing

We used the FMRIB Software Library (FSL v5.0.9; www.fmrib.ox.ac.uk/fsl)^71^ to process all DTI data. The preprocessing steps were identical to those of earlier work (for details, refer to Yang *et al*.^35^). First, we used the dcm2nii tool implemented in MRIcron (https://www.nitrc.org/projects/mricron/) to convert diffusion-weighted images from DICOM to NIFTI format. We used TOPUP^71,72^ and EDDY^73^ to reduce motion-related artifacts caused by susceptibility-induced distortions, eddy currents, and head movements. A single image without diffusion weighting (b0; b value = 0 s/mm^2^) was extracted from the concatenated data, and nonbrain tissue was removed using the FMRIB brain extraction tool (BET)^74^ to create a brain mask used for subsequent analyses. To derive the FA measure, DTIFIT^75^ was applied to fit a tensor model at each voxel of the data^71^.

To investigate the integrity of WM tracts, the tract-based spatial statistics (TBSS) implemented in FSL was applied^76^. FA images were slightly eroded, and end slices were zeroed to remove likely outliers from the diffusion tensor fitting. The images were then nonlinearly aligned (i.e., tbss_2_reg in FSL) to each other, and the most representative FA image was chosen. This target image was subsequently affine-transformed to 1-mm MNI space. FA images were transformed to 1-mm MNI space by combining nonlinear and affine registration. A skeletonization procedure was then performed on the group-mean FA image. The result was thresholded at FA > 0.2 to identify areas that most likely belonged to WM tracts.

### Fiber tract processing

After whole-brain TBSS, binary masks based on the probabilistic Johns Hopkins University (JHU) white-matter tractography atlas in FSL^77,78^ were created with a probability threshold of 5%. This tractography atlas allowed us to define white matter tracts and perform quantifications. To investigate the specific tracts within the frontal and parietal cortices, we adopted the atlas-based segmentation strategy. The superior longitudinal fasciculus (SLF) white matter tract was selected in each hemisphere defined by a previous study^79,80^. The underlying anatomical connectivity between the frontal and parietal cortices is thought to be mediated by the SLF, an associative white matter tract^81,82^. The average values of FA were computed for this SLF tract for each participant. Additional probabilistic tractography analysis was also conducted to derive FA values to ensure the validity of representative results of SLF tracts, and details are reported in Supplementary Methods S1 and Supplementary Tables S1–S4.

### GM volumetry

Regional GM volumes were estimated by FreeSurfer 5.3 (http://surfer.nmr.mgh.harvard.edu/)^83^ with an automated surface-reconstruction scheme described in previous well-established studies^84–87^. The recon-all flag -3T, a command implemented in FreeSurfer with an N3 bias field correction parameter, was deemed to be more appropriate for 3 T MRI^88^. Regions of interest (ROIs) were extracted using neuroanatomical labels in the Desikan-Killiany Atlas^89^ (https://surfer.nmr.mgh.harvard.edu/fswiki/CorticalParcellation) to map on a cortical surface model. GM volumes in each ROI of FreeSurfer’s atlas were extracted from output aseg.stats and aparc.stats files.

GM regions of interest, based on our initial hypothesis, were selected from the frontal lobe collapsed over the right and left hemispheres as defined by in the Desikan-Killiany Atlas^89^, which included the frontal pole (FPol), superior frontal (supF), rostral middle frontal (roMF), caudal middle frontal (cauMF), lateral orbital frontal (latOF), pars orbitalis (paORB), pars triangularis (paTriG), pars opercularis (paOPC), caudal anterior cingulate cortex (dACC), medial orbitofrontal (medOF) and rostral anterior cingulate cortex (rACC)^90,91^. The GM regions of interest in the parietal lobe spanned the right and left hemispheres and included the superior parietal (supP), inferior parietal lobe (IPL), postcentral gyrus (PoG), supramarginal gyrus (SMg), precuneus (Pc), posterior cingulate cortex (PCC), and isthmus cingulate cortex (ICC)^15,92,93^.

### Functional resting-state MRI preprocessing

In this study, functional images were preprocessed using CONN toolbox 18a (www.nitrc.org/projects/conn) and SPM 8 (http://www.fil.ion.ucl.ac.uk/spm) implemented in MATLAB (MathWorks, Inc., Natick, MA, USA). We also employed a recent preprocessing protocol^94^. The specific preprocessing steps were as follows: nuisance covariates including movement parameters (six parameters rigid body estimates) and averaged WM and CSF signals were regressed out after slice timing and motion realignment procedures. T1 images were first coregistered to the mean EPI image and then transformed to the MNI template. These coregistration parameters were applied to every functional volume. Additional frame-to-frame displacements at the subject level detected by “head motion censoring” (e.g., global-signal z-value threshold> 5 or the censoring of volumes whose framewise displacement (FD) power> 0.5 mm and one volume after the motion corrupted volume) were included as a covariate at the preprocessing level^95^. Then, a bandpass filter (0.008–0.1 Hz) was simultaneously applied to nuisance covariates and fMRI data^96^. Last, the functional data were spatially smoothed with an 8-mm Gaussian kernel.

### Functional connectivity (FC) analysis

The identification of large-scale functional networks was based on a Schäfer atlas^97^ that contains a cortical parcellation of 400 nodes of interest that are categorized into 17 functional networks^97,98^. This atlas has been shown to be more homogeneous^97^ than 4 previously published parcellations^99–104^. We utilized this cortical area parcellation (to be referred to as nodes) and selected only those nodes within the frontal and parietal lobules for the graph-theoretic analysis. Specifically, nodes of interest were defined using the anatomical mask for the GM analysis described in the GM volumetry section to select ROIs and those used for structural connectivity analysis. Graph-theoretic measures were further used to characterize each node’s topological properties^105^. Graph-theoretic metrics provide quantitative measures of network properties^106–108^, which allowed us to directly test the relationship between brain network properties and EFs. For each individual, we calculated two graph-theoretic brain network metrics related to the functional role of individual nodes within the frontal and parietal lobules. Specifically, we computed the participation coefficient (PC), a measure of internetwork connections and a within-module degree z-score (WMD), which is a measure of intranetwork connections^109^. PC and WMD were used to define different types of nodes that are important for network communication. PC is a network metric that indicates the distribution of a node’s connections across modules in a brain functional network, whereas WMD quantifies the degree to which a node is connected with other nodes within the same module. For example, if a node has a high WMD, then it can be considered a provincial hub; if it has a high PC, it is classified as a connector hub. Detailed methods of calculation and definition to estimate these components can be found in previous well-established studies^110,111^. PC and WMD were averaged across frontal-parietal nodes before being entered into the model. We only included nodes within the frontal and parietal lobules in our mediation model (see Supplementary Table S5 for coordinates of each node of interest) to examine the mediating role of these brain regions in age-related differences in EF.

### Statistical analysis

**Figure 7:**
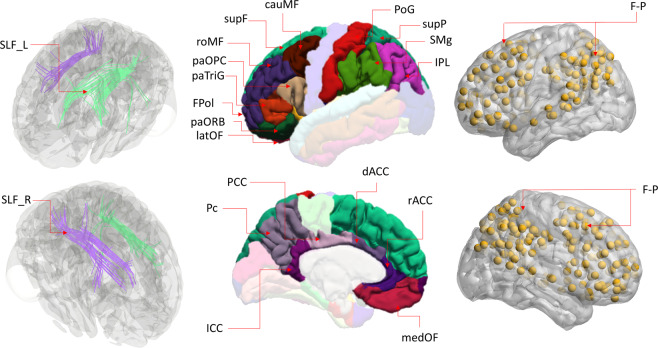
Eight structural and functional brain measures within frontal-parietal lobules. Left column indicates 2 white matter tracts, including SLF (L(left)/R(right), selected from diffusion tensor imaging maps (JHU white-matter tractography atlas) provided in the FMRIB Software Library (FSL); Middle column indicates the gray matter regions of the frontal and parietal lobe based on FreeSurfer-defined regions (Desikan-Killiany atlas); Right column indicates nodes for the functional connectivity networks of two matrices (i.e. participation coefficient, (PC) and within-module degree (WMD)), FPN, derived from the Yeo *et al*.’s (2011) network partitioning scheme. SLF = superior longitudinal fasciculus; F-P = fronto-parietal lobules, FPol = frontal pole, supF = superior frontal, roMF = rostral middle frontal, cauMF = caudal middle frontal, latOF = lateral orbital frontal, paORB = pars orbitalis, paTriG pars = triangularis, paOPC = pars opercularis, dACC = caudal anterior cingulate cortex, medOF = medial orbitofrontal, and rACC = rostral anterior cingulate cortex; supP = the superior parietal, IPL = inferior parietal lobe, SMg = supramarginal gyrus, PoG = postcentral gyrus, Pc = precuneus, posterior cingulate (pCC), isthmuscingulate.

This study tested the potential mediating effects of structural and functional brain measures within the frontal and parietal lobules on the relationship between age and Miyake and Friedman’s unity versus diversity of higher-level EF (i.e., common EF, updating-specific EF, and shifting-specific EF). Correlation and regression analyses were performed using SPSS v22 (IBM, Armonk, New York). Structural and functional brain measures within the frontal and parietal lobules included GM regional volumes, WM tract FAs, and intra/internetwork connection strength as calculated with PC and WMD scores. The procedures of the statistical analyses were as follows: (1) we first standardized all the variables (including independent, mediating, and dependent variables) into z scores using the data transformation function in SPSS and then examined the relationship between age and each EF score by conducting Pearson correlation and linear regression analyses in SPSS; (2) the correlation analyses were first performed among EF components, and then a correlation between age and each of the brain measures within frontal and parietal lobules was performed; and (3) mediation effects of brain imaging measures on the relationship between age (as an independent variable) and each of the three EF components were then examined, which resulted in a total of 3 mediation models. In the correlation analysis and each mediation model, gender, education level, and BDI-II scores for each individual were included as covariate variables to mitigate potential confounds. For the mediation analysis, we used Mplus version 8 ^112^ to build a mediation path model without any latent variables. This estimated both the direct and indirect effects on all three EF components (see Fig. 6c). The model was estimated using maximum likelihood estimation and bootstrapping methods^58^. The significance of indirect effects was assessed with a 95% confidence interval. To estimate confidence intervals, we used a bias-corrected method with the percentile bootstrap estimation approach, which ran 5000 bootstrap iterations that were implemented^59^. Adopting a two-tailed p < 0.05, we rejected the null hypothesis if the interval did not include zero. In particular, the rationale of the bootstrapping approach over other traditional approaches of mediation analysis is its improved sensitivity for estimating indirect effects^113^. To interpret the results, if the CI included zero, we concluded that the indirect effect was not significant because zero suggests no relationship between the mediator and dependent variable. Conversely, the CIs that did not include zero suggested that there was a significant relationship^40^. Standardized coefficients are reported after the data were transformed to z-scores and before entry into the model. In each of the 3 mediation analyses, all 8 brain measures (Fig. 7) were simultaneously entered into each of the models.

### Ethical statement

All of the experimental methods in this study were carried out in accordance with the Declaration of Helsinki and the rule of research in the University, and were approved by the Human Research Ethics Committee of the National Cheng Kung University, Tainan, Taiwan to protect the participants’ right. All participants signed the informed consent form before participating in the experiments.

## Supplementary information

Supplementary Tables 1–5.

## Data Availability

The authors confirm that the data supporting the findings of this study are available within the article and its supplementary materials. The data that support the findings of this study are available from the corresponding author, SH, upon request.
