# Supplementary material for: Frontoparietal structural properties mediate adult life span differences in executive function
Source: Sci Rep. 2020 Jun 3;10:9066. doi: 10.1038/s41598-020-66083-w (PMC7271169; doi:10.1038/s41598-020-66083-w)
Supplement: Supplementary file 1 — Supplementary Tables 1–5. [file 41598_2020_66083_MOESM1_ESM.pdf]

Supplementary Information

Frontoparietal structural properties mediate adult life span differences in executive function

Zai-Fu Yao, Meng-Heng Yang, Kai Hwang, Shulan Hsieh

Supplementary Methods S1

Probabilistic tractography analysis

The diffusion tensor (DT) was estimated on a voxel-by-voxel basis using the DTIfit toolbox, part of the FMRIB Diffusion Toolbox within FSL v5.0 (http://fsl.fmrib.ox.ac.uk/fsl/fslwiki/)[1], fitted diffusion tensors on eddy-corrected[2] data (http://fsl.fmrib.ox.ac.uk/fsl/fslwiki/EDDY). Probabilistic tractography analysis was followed procedures described in the (https://fsl.fmrib.ox.ac.uk/fsl/fslwiki/FDT/UserGuide). Bayesian fitting of the probabilistic diffusion model on eddy-corrected data using BEDPOSTx[3,4] followed by probabilistic tractography using PROBTRACKx2[3] implemented in FSL. Masks of the superior longitudinal fasciculus (SLF) white matter tracts was conducted in standard space of each participant using the seed/exclude masks on SLF from the AutoPtx plugin[5,6] for FSL. Fiber tracking was initiated from all voxels within the seed masks in the diffusion space to generate 5000 streamline samples with a step length of 0.5 mm and a curvature threshold of 0.2. We then take the output of tractography (i.e. waytotal and number of streamlines) transform to same anatomical space using fslmaths and fslstats to obtain tract-averaged measures of fractional anisotropy (FA) maps. To this aim, the number of streamlines present in the voxels of the tract maps was divided by the waytotal, which corresponds to the total number of streamlines that were not rejected by the exclusion masks. The tract masks obtained were thresholded at a value equal to 40% of the 95th percentile of the distribution of the intensity values of the voxels included in the tract. These results were then entered the mediation model reported in the Supplementary Tables S1-S4.

References:

1. Jenkinson, M., Beckmann, C. F., Behrens, T. E. J., Woolrich, M. W. & Smith, S. M. Fsl. *Neuroimage* **62**, 782–790 (2012).

2. Andersson, J. L. R. & Sotiropoulos, S. N. An integrated approach to correction for off-resonance effects and subject movement in diffusion MR imaging. *Neuroimage* **125**, 1063–1078 (2016).

3. Behrens, T. E. J., Berg, H. J., Jbabdi, S., Rushworth, M. F. S. & Woolrich, M. W. Probabilistic diffusion tractography with multiple fibre orientations: What can we gain? *Neuroimage* **34**, 144–155 (2007).

4. Jbabdi, S., Sotiropoulos, S. N., Savio, A. M., Graña, M. & Behrens, T. E. J. Model-based analysis of multishell diffusion MR data for tractography: How to get over fitting

problems. *Magn. Reson. Med.* **68**, 1846–1855 (2012).

5. Wakana, S., Jiang, H., Nagae-Poetscher, L. M., van Zijl, P. C. M. & Mori, S. Fiber Tract–based Atlas of Human White Matter Anatomy. *Radiology* **230**, 77–87 (2004).

6. De Groot, M. *et al.* Improving alignment in Tract-based spatial statistics: Evaluation and optimization of image registration. *Neuroimage* **76**, 400–411 (2013).

Supplementary Table S1. Correlation between age and probabilistic tractography results of the Superior longitudinal fasciculus (SLF) in left and right hemisphere. FA = fractional anisotropy; SLF = superior longitudinal fasciculus.

	age	
	r	*p*
FA		
SLF_L	-0.37	2.60 x 10^{-5}
SLF_R	-0.36	5.10 x 10^{-5}

Supplementary Table S2. Mediation path model of brain imaging measures in common EF. Standardized beta (β) estimates are shown for the relationship between age (A), each brain measure (B), and cognition (C). B∈k indicates the set of all brain measures. Asterisk measures indicate significance at p < .05 (direct effect) or bootstrapped 95% confidence intervals (indirect effects). The sample size was N = 126. SLF = superior longitudinal fasciculus; L = left; R = right; F-P = fronto-parietal lobules; PC = participation coefficient; WMD = within-module degree.

	β(A − Bk)	β(Bk − C)	β(A − Bk − C)
SLF_L	-0.395*	-0.213	0.084
SLF_R	-0.378*	0.188	-0.071
Frontal_L	-0.643*	-0.640*	0.411*
Frontal_R	-0.640*	0.583*	-0.373*
Parietal_L	-0.524*	-0.152	0.080
Parietal_R	-0.544*	0.227	-0.124
PC_F-P	-0.120	0.064	-0.008
WMD_F-P	0.057	0.009	0.001

	β (A − C)	β(A − B∈k − C)
age	0.186*	
age'	0.185*	0.001

Supplementary Table S3. Mediation path model of brain imaging measures in shifting EF. Standardized beta (β) estimates are shown for the relationship between age (A), each brain measure (B), and cognition (C). B∈k indicates the set of all brain measures. Asterisk measures indicate significance at $p < .05$ (direct effect) or bootstrapped 95% confidence intervals (indirect effects). The sample size was N = 126. SLF = superior longitudinal fasciculus; L = left; R = right; F-P = fronto-parietal lobules; PC = participation coefficient; WMD = within-module degree.

	β(A − Bk)	β(Bk − C)	β(A − Bk − C)
SLF_L	-0.395*	-0.336	0.133
SLF_R	-0.378*	-0.222	-0.084
Frontal_L	-0.643*	-0.197	0.127
Frontal_R	-0.640*	0.139	-0.089
Parietal_L	-0.524*	0.080	-0.042
Parietal_R	-0.544*	0.067	-0.037
PC_F-P	-0.120	0.080	-0.010
WMD_F-P	0.057	-0.147	-0.008
		β (A − C)	β(A − B∈k − C)
age		-0.223*	
age'		-0.213	0.010

Supplementary Table S4. Mediation path model of brain imaging measures in updating EF. Standardized beta (β) estimates are shown for the relationship between age (A), each brain measure (B), and cognition (C). B∈k indicates the set of all brain measures. Asterisk measures indicate significance at $p < .05$ (direct effect) or bootstrapped 95% confidence intervals (indirect effects). The sample size was N = 126. SLF = superior longitudinal fasciculus; L = left; R = right; F-P = fronto-parietal lobules; PC = participation coefficient; WMD = within-module degree.

	β(A − Bk)	β(Bk − C)	β(A − Bk − C)

SLF_L	-0.395*	-0.026	0.010
SLF_R	-0.378*	0.113	-0.043
Frontal_L	-0.643*	-0.280	0.180
Frontal_R	-0.640*	0.094	-0.060
Parietal_L	-0.524*	0.185	-0.097
Parietal_R	-0.544*	-0.085	0.046
PC_F-P	-0.120	0.162*	-0.019
WMD_F-P	0.057	0.122	0.007
		β (A − C)	β(A − B∈k − C)
age		0.296*	
age'		0.272*	0.024

Supplementary Table S5. The coordinates of FC ROIs within the frontal and parietal lobules.

Parcel_ID	MNI coordinates		
	x	y	z
64	47.9	-65.3	14.9
65	31.8	-84.1	27
66	26.4	-70.1	30.4
67	21.2	-78.6	44.7
68	22.7	-65.5	46.4
69	28.7	-58	50.6
70	35.9	-51.8	56
71	15	-71.1	57
72	28.2	-61.2	62.3
73	61.4	-23.3	32.8
74	54.8	-19.8	40.7
75	54.8	-31.3	44.4
76	46.2	-29.1	43.8
77	39	-37.5	49.8
78	30.2	-46.3	62.7
79	7.4	-58.4	63
80	19.7	-56.5	66.2
81	12.8	-51	70.9
82	40.3	-2.3	51.3
83	25.7	-0.5	55.2
84	30	-8	51.7

85	50	2.6	38
86	54.6	-32	22.2
87	58	-44.2	27.3
88	61.2	-36.1	33.3
89	38.7	1.9	-4.6
90	39.8	-14.4	-1.7
91	33	19.2	8.2
92	36.5	4.4	10.7
93	50.5	1.5	4.4
94	52.1	8.7	13.3
95	11	-27.4	41.1
96	12.6	-41.5	47.3
97	5.8	-49.1	57
98	6.5	0.8	40.5
99	5.3	9.3	48.4
100	6.7	-3.8	68
101	38.2	49	11
102	28.9	43.3	29.5
103	35.9	32	38.5
104	33.4	16.5	-8.3
105	32.9	24.6	-0.8
106	43.3	11.9	2.3
107	27.1	49.3	-13.7
108	6	21.8	31.1

109	12.4	24.3	-20.3
110	23.6	22.4	-19.7
111	10.6	47.3	-21.3
112	3.9	23.5	-18.9
122	28.8	-74.2	42.3
123	58.3	-41.6	45.6
124	34.4	-62.1	47.9
125	45	-41.3	46.7
126	33.1	-46.5	40.9
127	21.3	5.5	63.2
128	47.9	35.1	10.5
129	41.6	37.7	21.9
130	49.4	6	25.6
131	44.4	20.2	27.2
132	39.2	7.4	33.5
133	3.3	6.4	28.5
136	48.9	-59.5	46.9
137	53.2	-49.6	44.8
138	42.1	-52.2	48.6
139	29.9	13.6	57.2
140	41.9	49.1	-6.5
141	28	58.4	-0.7
142	27.6	56.8	13
143	3.7	28.1	47.1

144	10.3	-69.6	31.3
145	9.1	-77.1	45.3
146	5	-63.8	51.9
147	5.7	-40.6	23.3
148	4.1	-21.7	28.9
149	47.3	-63.6	31.3
150	41.4	-72	43.3
151	24.8	28.3	43.5
152	18.4	35.8	48.1
153	22.4	19.4	51.8
154	4.2	-53.4	20.2
155	5.3	-60.2	30.1
156	7.4	-44.3	32
157	4	-33.9	37.8
158	2.6	-15.5	37
159	3	-68.2	41.2
160	6.7	-50.9	42.8
161	4.7	55.1	-10.1
162	5.9	34.9	-8.8
163	6.4	59.2	6.5
164	6.1	44.7	5.9
165	16.2	67	8.4
166	5.4	33.7	20.4
173	45.7	-59	21.1

174	56.5	-54.6	29.7
175	4.2	51.1	27.8
176	13.8	57.5	30.9
177	21.9	50.2	31.3
178	8.1	42.3	51.3
179	12.5	24.3	60.9
180	6.1	10.7	63
181	41.3	19	47.8
182	41.6	7.1	48.6
183	35.6	22.3	-15.5
184	35.9	36.9	-13
185	46.2	31.6	-10.3
186	47.7	28.3	0.5
187	53	19.2	11.5
188	40.4	-78.5	30.2
195	52.8	6.2	-11.6
196	60.5	-12.6	-2.7
197	61.7	-31.9	5
198	52.6	-43	5.1
199	56.6	-53.7	10
200	58.5	-49	16.2
264	-35.6	-79.3	24.2
265	-28.9	-77.7	36.7
266	-31.6	-66.5	35.4

267	-18.4	-79	50.1
268	-30.7	-63.8	52.7
269	-20.8	-68.6	52.9
270	-33.8	-50.6	54.1
271	-26.6	-58.1	60.6
272	-13.5	-64	64.8
325	-35.1	-70.7	46.5
326	-53.3	-33.3	50.6
327	-46.8	-44.1	46.6
328	-36.3	-44.8	44.9
329	-24.1	9.9	57.4
330	-48.4	30.2	18
331	-48	18.1	23.5
332	-46.5	28.8	27.6
333	-48.8	8.2	24.7
334	-39.3	10.7	33.8
335	-4.8	1.4	30.3
338	-55.6	-45.2	32.4
339	-54.3	-52.7	44
340	-55	-40.5	47.1
341	-40.9	-55.1	48.4
342	-38.8	33.3	37.8
343	-43.9	18.6	43.2
344	-42.7	5.9	50

345	-33.9	15.6	55.3
346	-35.1	37.7	-13.7
347	-27.9	54.8	-13.5
348	-42	50.8	-6.4
349	-27.4	59.1	2.7
350	-4.8	28.3	48.1
351	-16.5	-63.4	27.5
352	-13.1	-71.5	39
353	-5.7	-64	43.6
354	-7.2	-49.6	44.4
355	-8.5	-71	52.4
356	-7.3	-43.7	19.8
357	-6.4	-26.3	27.9
359	-53	-53.3	25.9
360	-47.3	-64.3	41.6
361	-26.2	34.5	39.3
362	-23.9	25.6	50.5
363	-5.8	-52.2	22.9
364	-4.9	-63.3	31.3
365	-6.7	-38.8	34.8
366	-4.1	-19.7	37
367	-9.9	-52.6	35.1
368	-5.4	40.4	-10.2
369	-9.4	66.6	1.1

370	-7.6	42	4.2
371	-7.4	54	12.8
372	-17.5	64.8	16.1
373	-5.7	24.8	18.2
374	-62.7	-23.2	-7.5
375	-62.9	-37.4	0
377	-6.3	57.5	29.4
378	-16.3	52.5	35.7
379	-4.8	44.5	40.1
380	-13.9	38.7	52.2
381	-12.1	20	62.3
382	-35	23	-17.2
383	-47.8	31.4	-7.8
384	-52.7	23.5	6.2
385	-48.1	-64.1	21.7
386	-45.2	-75.1	30.5
391	-47.5	16.1	-20.1
392	-54.7	-4.1	-14.2
393	-49	-20.1	-7.4
394	-61.5	-18.6	-0.5
395	-50.4	-33.1	1.5
396	-59	-46.3	6.9
397	-51.8	-40.8	12.2
398	-63.9	-34.6	10.2

399	-54.1	-45.6	19.8
400	-60.9	-39.6	21.8
